# Coloration Phenomenon of Flunitrazepam on the Tongue of an Elderly Patient: A Case Report

**DOI:** 10.7759/cureus.60627

**Published:** 2024-05-19

**Authors:** Ryo Ichibayashi, Miwako Togawa

**Affiliations:** 1 Division of Emergency Medicine Department of Internal Medicine, Toho University Medical Center Sakura Hospital, Chiba, JPN; 2 Division of Emergency Medicine, Department of Internal Medicine, Toho University Medical Center Sakura Hospital, Chiba, JPN

**Keywords:** prohibited drugs, tongue, coloration phenomena, elderly, flunitrazepam

## Abstract

Patients with dementia may forget to take their oral medications or may accidentally take too much. Furthermore, there are cases where people lick the medicine without recognizing it as a medicine or accidentally ingest it.

An 88-year-old woman with a history of insomnia presented to the hospital, complaining of her weakness and mild loss of consciousness. Although her blood tests, imaging studies, and neurological findings were unremarkable, we noticed that her tongue was blue and determined that she had mistakenly taken flunitrazepam. This accidental ingestion was diagnosed as the cause of the symptoms.

Patients with dementia report that they may take medicine by licking it, and some oral medicines have a coloring effect.

## Introduction

As we get older, we usually tend to take more prescription drugs. Declining cognitive function in older adults increases the risk of forgetting to take medications, overdosing, or taking them at the wrong time [1].

If you take tablets or capsules without water, they may stick to your mouth or esophagus. If they melt on the spot, they may cause esophageal ulcers [2]. Therefore, if your cognitive function has deteriorated, you need to be careful about how you take your medication.

Our case presentation is of a patient with dementia who mistook oral medication for candy and ingested it. We report that patients with dementia may take medication in this way and that some drugs can color the oral cavity, depending on the type of tablet.

## Case presentation

An 88-year-old woman with a medical history of insomnia and dementia, who lived alone, was admitted by her family due to dysarthria and weakness. The medication she took regularly was a sleeping pill. Her vitals were as follows: Glasgow Coma Scale score of 14, blood pressure of 99/65 mmHg, pulse rate of 67 beats per minute, SpO2 of 100% on room air, body temperature of 36.1 °C, and pupil diameters of 2.12 mm on the right and 1.78 mm on the left. Bilateral light reflexes were observed, with measurements taken using a quantitative pupillometer. She had slurred speech and disorientation but no other neurological abnormalities. Blood tests revealed no abnormalities in ammonia or electrolytes (Table 1).

**Table 1:** Laboratory results upon arrival. CRP, C-reactive protein; TP, total protein; Alb, albumin; AST, aspartate aminotransferase; ALT, alanine aminotransferase; LDH, lactate dehydrogenase; ALP, alkaline phosphatase; γ-GTP, γ-glutamyl transpeptidase; T-Bil, total bilirubin; BUN, blood urea nitrogen; eGFR, estimated glomerular filtration; HbA1c, hemoglobin A1c

Test	Result	Unit	Reference range
Ammonia	30	mg/dL	30-86
CRP	0.04	mg/dL	<0.3
TP	7.0	g/dL	6.7-8.3
ALB	3.8	g/dL	3.8-5.2
AST	26	IU/L	10-40
ALT	19	IU/L	5-45
LDH	185	U/L	124-222
ALP	43	U/L	38-113
γ-GTP	10	IU/L	<30
T-Bil	0.6	mg/dL	0.2-1.2
BUN	20.1	mg/dL	8.0-20.0
Creatinine	0.63	mg/dL	0.47-0.79
eGFR	66	mL/minute/1.73 m^2^	
Sodium	156	mEq/L	137-147
Potassium	3.5	mEq/L	3.5-5.0
Chlorine	115	mEq/L	98-108
Calcium	12.7	mg/dL	8.4-10.4
IP	2.9	mg/dL	2.5-4.5
Magnesium	2.8	mg/dL	1.9-2.5
Glucose	119	mg/dL	70-109
HbA1c	5.3	％	4.6-6.2

**Figure 1:**
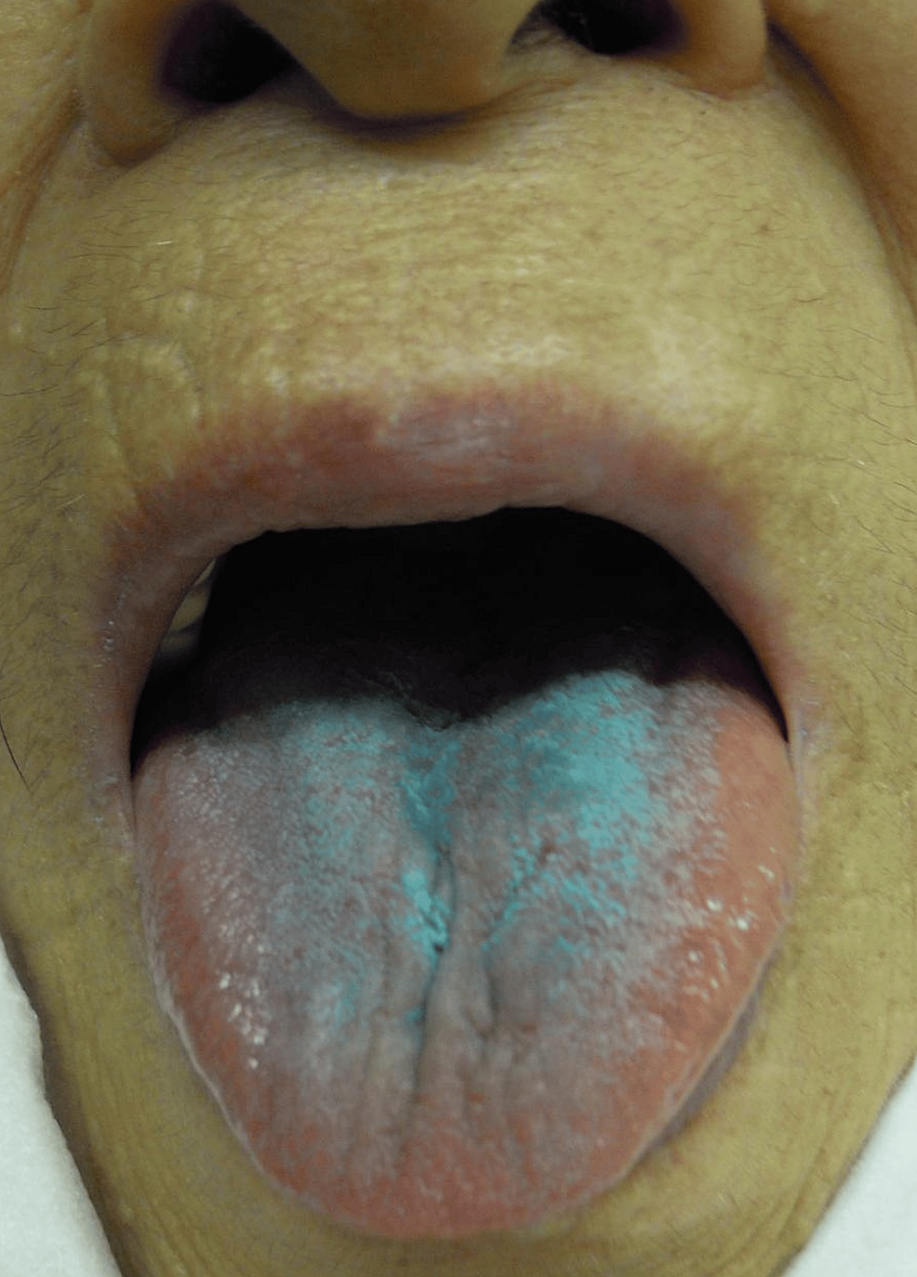
The tongue is stained blue.

The electrocardiogram showed sinus rhythm and no QTc prolongation. Head computed tomography (CT） revealed no bleeding. A head magnetic resonance imaging (MRI) revealed only atrophy of her brain and no other abnormal findings. However, upon repeated examination, we noticed that her tongue had turned blue (Figure 1).

We thought that she might have taken some drugs. Therefore, we added an abdominal CT scan to evaluate the gastric contents. No pills or contents were found in the stomach. After inquiring about her medication intake, her family revealed that she had taken her medicine by licking it like candy on the morning of her transportation. The drug turned out to be flunitrazepam, and we concluded that it was the cause of the blue discoloration on her tongue. Therefore, the cause of her dysarthria and weakness was diagnosed as the effects of flunitrazepam. Her score on the Revised Hasegawa Dementia Scale (HDS-R) was 15 points, indicating the possibility of dementia. The symptoms improved after six hours of infusion and observation at the outpatient clinic. She was referred to a psychiatric clinic for treatment of her dementia.

## Discussion

This is an example of the importance of observing the inside of the oral cavity and knowing the presence of dye-containing tablets when taking oral medicine by licking it like candy. Flunitrazepam is a benzodiazepine sleep-inducing drug that is misused as a date rape drug in the United States. For this reason, in 1997, the tablets were changed to green rectangles in the United States, and improvements were made to make them blue when mixed with liquid [3]. In Japan, there are many suicide overdoses, so in 2015, a blue dye was incorporated into the tablets. The pills were designed to release the blue dye when crushed and dissolved in liquid. When taken orally, it turns blue in the stomach due to gastric juices. If it disintegrates in the mouth and remains there for a certain period, it may cause the mouth's tongue and inside to turn blue.

Patients with dementia and children are more likely to take medications incorrectly. Not only are there problems with dosage, such as forgetting to take medication or overdosing, but there are also cases of accidentally ingesting medicines in blister packaging or Press Through Packaging (PTP). Therefore, this coloring phenomenon may occur in patients with dementia and children who may take the drug incorrectly. For this reason, it is important to know that patients with dementia and children may take the drug by licking it like candy while keeping it in their mouth and that there are drugs that become colored when they react with body fluids such as saliva and gastric juices [4]. This will help detect accidental ingestion.

Chest discomfort and chest X-ray can assist in the diagnosis of accidental ingestion of PTP. Intraoral observation has become a diagnostic aid for atypical symptoms, such as in this case. Traditionally, when gastric lavage is performed in poisoning treatment, the inside of the oral cavity is observed. However, in modern times, where the effectiveness of gastric lavage is uncertain and its use in treating poisoning is decreasing, the probability of noticing abnormalities in the oral cavity may also be decreasing [5].

## Conclusions

When treating geriatric patients, it is important to consider the effects of drugs on patients with weakness or impaired consciousness. When the drug is flunitrazepam, intraoral observation is important as it assists in medical diagnosis and treatment. The same can be said for other drugs that dissolve in liquids and produce color tones.
